# Acute aerobic exercise benefits allocation of neural resources related to selective attention

**DOI:** 10.1038/s41598-023-35534-5

**Published:** 2023-05-27

**Authors:** Tomasz S. Ligeza, Marie Julie Vens, Thea Bluemer, Markus Junghofer

**Affiliations:** 1 Psychophysiology Laboratory, Institute of Psychology, Jagiellonian University, Ingardena 6, 30060 Kraków, Poland; 2 Institute for Biomagnetism and Biosignalanalysis, University of Muenster, Muenster, Germany; 3 Otto Creutzfeldt Center for Cognitive and Behavioral Neuroscience, University of Muenster, Muenster, Germany

**Keywords:** Neuroscience, Psychology

## Abstract

A single session of aerobic exercise has been shown to potentially benefit subsequent performance in a wide range of cognitive tasks, but the underlying mechanisms are still not fully understood. In this study, we investigated the effects of exercise on selective attention, a cognitive process that involves prioritized processing of a subset of available inputs over others. Twenty-four healthy participants (12 women) underwent two experimental interventions in a random, crossover, and counterbalanced design: a vigorous-intensity exercise (60–65% HRR) and a seated rest (control) condition. Before and after each protocol, participants performed a modified selective attention task that demanded attending stimuli of different spatial frequencies. Event-related magnetic fields were concurrently recorded using magnetoencephalography. The results showed that exercise, relative to the seated rest condition, reduced neural processing of unattended stimuli and increased processing of attended stimuli. The findings suggest that changes in neural processing related to selective attention may be one of the mechanisms underlying exercise-induced improvements in cognition.

## Introduction

A single aerobic exercise session (acute exercise; hereafter, “exercise”) is associated with subsequent benefits in cognitive functions^1,2^. The evidence to support this relationship is based mainly on improved performance in complex tasks that rely on several cognitive operations, including attention to stimuli, decision-making, or responding^3^. As these processes are not easily separable, their individual contributions to exercise-induced benefits are still poorly understood. We here aimed to verify the effects of exercise specifically on selective attention—a process of prioritized processing of a subset of available stimuli over other inputs. Compared to previous research, we used a task that was aimed to better isolate the selective attention process from other higher cognitive operations and measured the allocation of neural resources to stimuli that demand different levels of selective attention.

The effects of exercise on cognition are most often tested using tasks assessing executive function (cognitive control)^1,2^. Executive functions involve a set of higher-order cognitive operations that regulate goal-directed behavior^4^. They mainly include inhibition (ability to inhibit irrelevant stimuli, thoughts, or behaviors), working memory (ability to hold information in one's mind and manipulate it accordingly to goals), cognitive flexibility (ability to switch task goals adaptively), and executive attention (ability to control attention accordingly to goals). The most popular tasks testing executive functions are the flanker task, the Stroop task, the go/no-go task (inhibition/executive attention), and the n-back task (working memory). In addition to testing specific executive functions (in a specific task condition, e.g., as assessed by the difference in reaction times/accuracy between congruent and incongruent trials in the Stroop task), all these tasks rely on general cognitive processes related to stimuli evaluation, decision-making, and responding.

Two main hypotheses describe the nature of the effect of exercise on executive functions. The "general improvement hypothesis" indicates that exercise benefits general task performance, i.e., overall response times or accuracy, irrespective of task conditions. In contrast, the "selective improvement hypothesis" suggests that exercise benefits specific executive functions like inhibition. Although both hypotheses are confirmed by research, increasing evidence shows that exercise predominantly benefits general task performance^1,2,5–7^. For example, two meta-analyses, which collectively analyzed 40 experimental studies investigating the acute effects of exercise on specific aspects of executive functions^1^ and 80 long-term exercise interventions^7^, suggest a general effect of exercise on cognition rather than a domain-specific effect. This suggests that exercise might boost some of the general cognitive processes shared among all executive functions. As these processes are not easily separable, little is known about the effects of exercise on more fundamental cognitive processes.

One of the most fundamental cognitive processes associated with stimuli evaluation is so-called selective attention: attentional resources assigned to a given stimulus might determine the course of the other cognitive processes. Yet, the influence of exercise on the very process of selective attention is still poorly understood. Many studies have investigated the effects of exercise on executive attention. Notably, however, there is a significant difference between executive attention and selective attention. Selective attention and executive attention are related but distinct processes. Executive attention is a broader concept encompassing various attentional processes involved in goal-directed behavior, while selective attention specifically refers to the ability to focus on specific stimuli while filtering out irrelevant information. Selective attention refers only to the process of stimuli evaluation, a process that is also driven automatically^8,9^. This process is also closely related to perception and sensory processing. Executive attention, in contrast, refers to the ability to control and direct attention consciously based on one’s goals, intentions, and task demands. It involves higher-level cognitive processes, such as goal-setting, decision-making, and cognitive control. The tasks used in research so far have not been able to isolate the very process of selective attention. This applies to the most frequently used tasks in the exercise paradigm, like the flanker^10^ and Stroop tasks^11^ (which directly tests executive attention), but also to tasks testing other aspects of attention, such as orienting or spatial attention (allocating attention to a particular object in space)^12^ or alerting attention (preparing the perceptual and motor system for fast reactions)^13^. The exercise-induced improvements in the above tasks might reflect not only processes of selective attention but also higher-order processes of directing attention according to the task aim, refraining from incorrect motor responses, deciding on how to react, and implementing reactions.

Given the multitude of processes in popular cognitive tasks, some studies have utilized neural measures to understand the effects of exercise on more fundamental cognitive processes. The most popular neural method to serve this aim is the measure of event-related potentials (ERPs). Derived from the neuroelectric system (EEG), ERPs are voltage changes recorded from the scalp with temporal coincidence to events^14^. Their high temporal resolution is particularly well suited to providing insights beyond what may be understood through overt behavioral measures. The most popular and conspicuous ERP component is the so-called P3b (P300), a positive-going deflection occurring 300–700 ms after stimulus presentation^15^. An increase in P3b amplitude during executive functions/attention tasks is the most consistent finding for exercise-induced ERP changes; a meta-analysis showed that 19 of 23 analyzed studies demonstrated acute modulation of P3b following exercise cessation^16^. According to the mainstream view, the P3b amplitude is hypothesized to be sensitive to the allocation of attentional resources such that the amplitude is proportional to the amount of resources allocated toward task-relevant stimuli in later, post-perceptual stages of cognitive processing^17^. Based on this interpretation, acute exercise has been proposed to benefit the allocation of attentional resources, which is also suggestive of the influence on selective attention^18–20^.

Several studies have considered the effects of exercise not only on P3b but also on earlier ERP components such as N1 or P2, which may be markers of earlier (perceptual) selective attention^21–26^. However, these studies showed a significant effect of exercise only on the P3b but not on preceding components^21–23^ or found no significant effect of exercise on any of the analyzed ERP components^24^. Instead, significant effects of exercise on earlier ERP components were shown during rather than after exercise. For example, besides influencing P3b, exercise was found to affect N1, P2^25^, and P1 components ^26^ during an attentional task, suggesting multiple stages of information processing might be affected during exercise. However, whether these early effects persist after exercise remains unclear, as various physiological and psychological factors, such as changes in heart rate, blood pressure, and physiological arousal, may differ between the exercise and post-exercise states. Studies focusing on post-exercise assessment suggest that exercise primarily affects later stages of cognitive processing, marked by the P3b component. Yet, despite extensive research on the P3b, the functional significance of the P3b component has remained a contentious topic of debate. Increasing evidence is suggesting that the P3b component may not solely reflect attentional resource allocation but may play a role in decision-making or responding^27^.

Therefore, despite the suggestive evidence, the relationship between exercise and selective attention warrants further investigation. First, inferring about selective attention processes is challenging based on the tasks used in research so far. Second, neural evidence to suggest exercise-induced changes in attentional resource allocation is mostly based on the P3b component, whose exact functional meaning is unclear. We here aimed to provide more direct evidence on the exercise–selective attention relationship. First, we used a task that isolated selective attention from higher cognitive processes to the extent possible. Second, we use inverse models of distributed brain measures that provide more precise information about the location of brain activity. Below we present more details that distinguish our study.

**Figure 1:**
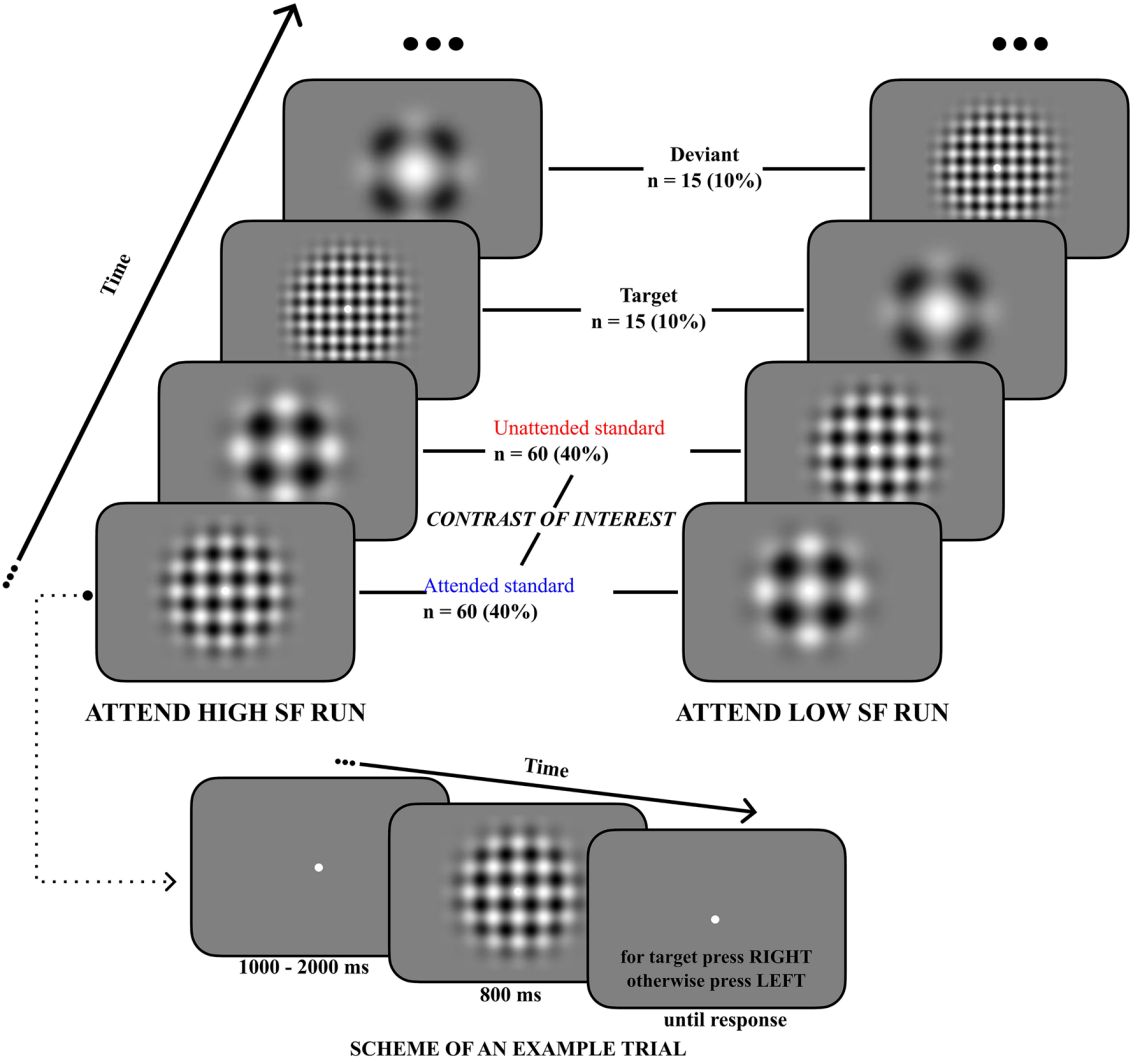
Schematic illustration of the selective attention task used in the study. Participants were presented with high and low spatial frequency (SF) Gabor gratings in each of the two runs. In an "attend high SF run," participants detected targets of slightly higher SF than corresponding high SF standards. In an "attend low SF run," participants detected targets of slightly lower SF than corresponding low SF standards. Detecting targets of particular SF was assumed to demand directing more attentional resources to standards of corresponding SF (attended standards) than for standards of distinct SF (unattended standards). The difference between attended and unattended standards was the main contrast of interest in the neural data analyses. Each trial started with a fixation dot followed by the stimulus and an instruction to respond. Participants responded with one finger to targets and another to non-target stimuli.

We employed a task that allowed us to manipulate attention allocation to visual stimuli with minimal involvement of other cognitive processes. Most importantly, we aimed to separate selective attention from executive attention by providing a task requiring participants to attend to a specific feature of the presented stimuli without explicit instruction to attend to them. The visual stimuli consisted of Gabor gratings that differed in spatial frequencies (SF). Standard gratings of high and low SF were presented frequently, along with rare targets/deviants of slightly higher or lower SF than the above SF standards. All stimuli were presented at the center of gaze, and participants aimed to detect one type of target in each of the two task runs (see Fig. 1 for schematic illustration). Specifically, they were instructed to detect targets with slightly higher SF than the high SF standards in one run and targets with slightly lower SF than the low SF standards in the other run. The central assumption behind the task is that participants must attend more to standards of corresponding SF (hereafter, “attended standards”) than to standards of dissimilar SF (“unattended standards”)when detecting the targets^28^. For example, detecting a high SF target requires directing more attentional resources to high SF standards than low SF ones. Thus, unlike classic oddball tasks that test the effects of distractors and targets, we focused on examining the pure effect of directed attention on the standards, independent of target or distractor processing and, notably, independent from the explicit instruction to detect targets (which is typical for executive attention). As selective attention is closely related to sensory processing, we instructed participants not to respond until the stimuli offset to further isolate the process of selective attention.

We measured the brain responses to attended vs. unattended standards using the event-related fields (ERF) of whole-head magnetoencephalography (MEG). ERFs, similar to ERPs, measure brain responses to events with the utmost temporal precision. However, instead of assessing the electrical activity of neurons, ERFs reflect their corresponding magnetic fields^29^. The magnetic fields, in contrast to the electric currents, pass through the head with almost no distortion, allowing more accurate source modeling. This allowed us to focus on brain regions that are specifically related to processes of attention to perceptual stimuli. According to the literature, the effects of attention manipulation (attended vs. unattended stimuli) were expected to be located in the posterior visual cortex regions of the brain, predominantly in the extrastriate visual areas^28,30,31^. The onset of this effect was expected to start relatively early (approx. 100–200 ms after stimuli onset), representing neural activity related to selective attention.

Given the research showing that exercise influences cognition and, presumably, attentional processing ^1,2,16^, exercise was expected to influence brain activations to attended vs. unattended stimuli. In particular, the difference in neural activation between attended and unattended stimuli was expected to increase after exercise due to increased responses to attended standards, decreased responses to unattended standards, or both. A control condition of seated rest was expected to have either no effect or a negative effect on the neural responses to attended vs. unattended stimuli (i.e., relatively decreased/increased neural activations in response to attended/unattended stimuli after rest). While the majority of studies reveal no significant impact of a seated rest condition on cognitive measures, a subset of them does suggest potential detrimental effects ^32–34^.

Overall, we aimed to obtain evidence for (or against) the current theories explaining the relationship between exercise and cognitive performance. Confirming hypotheses would support the previous findings about the role of attention allocation in exercise-induced cognitive benefits. Although our procedure was optimized to reveal neural effects, in line with the previous literature, we expected that more accurate responses to target stimuli would accompany neural modulations. Due to the intentional lack of time pressure, we did not analyze reaction times.

## Methods

### Participants

Twenty-eight Caucasian adult participants were recruited via advertisements posted on local online advertising services, newsletters, and social media portals of the University of Muenster, Germany. Inclusion criteria were as follows: 18–40 years old, normal or corrected-to-normal vision, no history of neurological disorders or substance dependence, being vaccinated for COVID-19, testing negative for COVID-19 infection, not having non-removable metallic objects on the body and not having exercise contraindications (as assessed by Physical Activity Readiness Questionnaire, PAR-Q). Based on our previous studies on the influence of exercise on brain processes^35,36^, we aimed to analyze clean data from 24 participants. One participant was excluded due to technical problems with data collection, and three participants were excluded due to analysis problems, such that the data of 24 participants (12 females/12 males) could be included in the final analyses. The participants’ characteristics are presented in Table 1. Each participant provided informed written consent. The experimental procedure complied with the directives of the Helsinki Declaration and was approved by the ethical board of the University of Muenster (Germany). Participants were instructed to avoid any physical activity for 24 h prior to each experimental visit and to abstain from food intake for 2 h prior to each visit. For measurement of physical activity, all participants completed the BSA Questionnaire by Fuchs^37^. The questionnaire consists of three scales: 1) work and leisure time activity (e.g., working as a postman, walking or cycling to work/school, climbing the stairs, gardening, housework, etc.); 2) sport activity; 3) total activity [1) + 2)]. Participants reported on activities they had been regularly engaged in within the last four weeks, providing the approximate time spent on these activities (frequency and duration in minutes). For each scale, we calculated a total index value in “minutes per week” (see: Table 1).

**Table 1:** Participant characteristics (M ± SD).

	Women	Men	Total
*N*	12	12	24
Age (years)	24.25 ± 2.60	24.50 ± 3.56	24.38 ± 3.05
Height (cm)	172.25 ± 7.60	183.17 ± 7.38	177.71 ± 9.20
Weight (kg)	64.92 ± 10.97	81.92 ± 12.01	73.42 ± 14.24
BMI (kg/m^2^)	21.83 ± 3.01	24.30 ± 2.48	23.07 ± 2.98
VO2peak (mL·kg^−1^·min^−1^)a	48.56 ± 6.45	43.90 ± 4.75	46.40 ± 6.18
WLTPA* (min/week)	437.93 ± 428.79	310.73 ± 221.62	371.57 ± 335.19
SPA* (min/week)	136.15 ± 96.11	224.48 ± 152.99	180.31 ± 132.84
TPA* (min/week)	586.46 ± 429.56	535.20 ± 285.23	559.72 ± 353.89
HR Rest (beats/min)	59.58 ± 5.99	59.08 ± 6.52	59.33 ± 6.13

*WLTPA* Work and leisure time physical activity, *SPA* Sport physical activity, *TPA* total physical activity (WLTPA + SPA), *BMI* Body mass index, *VO2peak* Maximal oxygen uptake, *HR* *Rest*  mean resting heart rate (measured after waking up), *as measured by the BSA Questionnaire (see Participants section for more details).

### Selective attention task

The experimental task consisted of a modified selective attention task as proposed by Martínez and colleagues^28^. In the task, participants attended to either high or low standard spatial frequency (SF) Gabor stimuli, with the aim to detect target stimuli with slightly higher or lower SF, respectively, than the above standards. (see Fig. 1 for an example stimulus and task overview).

#### Stimuli

Stimuli were circular checkerboard patterns with a sinusoidal progression of color changes between black and white in both horizontal and vertical directions (Gabor stimuli). The color intensity of a stimulus follows a Gaussian distribution and decreases toward the edges. Four types of stimuli that differed in terms of SF were used: 1) “lower SF standards”—checkerboards with SF of 0.5–0.8 cycles per degree (cpd); 2) “lower SF targets/deviants”—checkerboards with slightly (see below description for more details) lower SF than “lower SF standards”; 3) “higher SF standards”—stimuli with SF of 1.5–2.8 cpd; 4) “higher SF Targets/deviants”—checkerboards with slightly higher SF than “higher SF standards.” The standard stimuli (1,3) were presented four times more frequently (*P *= 0.80) than targets and deviants (3,4) (*P *= 0.20). Depending on the task’s run (“attend low”/ “attend high”; see description below), the infrequent stimuli were either targets or deviant stimuli.

#### The aim of the task

The aim of the directed attention task was to manipulate neural attentional resources to perceptually identical stimuli. The task consisted of two runs, in which participants attended to either “low SF” or “high SF” stimuli. In both runs, all of the four types of stimuli were presented. In the “attend low SF” run, participants were asked to detect the “lower SF targets” among the three remaining types of stimuli (higher/lower SF standards, higher SF deviants). In the “attend high SF” run, participants were asked to detect the “higher SF targets” among three remaining types of stimuli (higher/lower SF standards, lower SF deviants). As low and high SF standards were either attended or unattended in both runs, comparing attended and unattended stimuli across both runs thus reflected neural responses to perceptually identical stimuli. Participants had to place the index fingers of each hand on the response boxes and were asked to indicate by button press whether the presented stimulus was a target of the attended SF (reaction with one hand) or any other stimuli (reaction with the other hand). Note that in the original study by Martinez^28^, participants only responded to targets of attended SF, while they were not responding to the other types of stimuli. With a focus on neural effects, we here additionally asked participants to react to all of the incoming stimuli. This way, we aimed to avoid any data contamination driven by response preparation, which was expected to differ between attended standards and unattended standards. Please refer to the Supplementary Materials for an overview of further changes in task parameters between the current study and the original study .

The “attend low SF” run aimed to increase the involvement of attentional resources toward all stimuli of low SF (standards and targets alike) as compared to all high SF stimuli (standards and deviants alike). This is because the participants had to differentiate between lower SF targets and lower SF standards, whereas differentiation between higher SF deviants and higher SF standards was unnecessary in this run. Analogously, the “attend high SF” run aimed to increase the involvement of attentional resources toward high SF stimuli compared with low SF stimuli. Again, this is because the participants had to differentiate between higher SF targets and higher SF standards, whereas differentiation between lower SF deviants and lower SF standards was unnecessary. Importantly, this task aimed to compare neural resources dedicated to the low or high SF standards independent of the identification of the target stimuli or perception of the deviants. Thus, the processing of the target and deviant stimuli were not taken into consideration, and the 2 × 60 standard stimuli of the attended and unattended conditions only were considered in each run. As event-related fields evoked by low SF and high SF Gabor stimuli strongly differ (even though the stimuli were isoluminant and black and white), each participant had to attend to both low SF and high SF in two different consecutive runs. This way, the attended low SF and attended high SF standard stimuli of both runs could be merged and could be compared to the merged unattended low SF and unattended high SF standard stimuli of both runs. Due to potentially slightly divergent positions of the participant’s head in the MEG scanner between the two runs, we did not merge the event-related magnetic fields of both runs but merged the estimated neural activities (see below) of both runs, which are independent of the individual head position. Brain responses to stimuli of attended vs. unattended frequencies across both runs thus constitute the main dependent variable, reflecting the involvement of neural attentional resources of selective attention.

#### Task presentation parameters

The task was presented using the Psychophysics Toolbox^38^ in MATLAB (The MathWorks; Version R2018b). Each trial started with the presentation of a gray screen with a fixation dot at the center of the screen for a jittered time of between 1 and 2 s. Then, the Gabor stimuli were presented at the center of the screen for a duration of 800 ms. Afterward, the instruction to press the appropriate button appeared on the screen until a button press was recorded (see Fig. 1 for an example trial). Participants were not encouraged to respond as quickly as possible, and participants could not respond before the stimulus offset. The task was projected onto a screen (450 × 252 mm) located in the participants' chamber (PROPixx Lite, VPixx) via a 1920 × 1080-pixel projector at a viewing distance of approximately 90 cm, depending on the individual’s sharpness optimum.

The assignment of the response reaction with either the right or left index finger was balanced across participants (i.e., half of the participants used the right index finger for targets and the left index finger for all other stimuli and vice versa for the other half of participants). However, to avoid confusion, the assignment was kept identical for each participant across sessions (exercise, seated rest) and runs (attend low SF, attend high SF). All participants went through 50 practice trials before starting the main experiment, which contained a total of 300 trials in two runs (each run contained 150 trials, 60 low and 60 high SF standards, 15 targets, and 15 deviants). The spatial frequencies for attended and unattended standards remained at constant values, while the frequencies of targets and deviants were modulated during the runs. For the practice trials, the frequency differences between standards and targets or deviants, respectively, were significant enough to be detected by each individual. During the actual test procedure, the frequency differences were adjusted via the Quest function of the PsychoPhysics toolbox based on the individual participant’s performance. The frequency difference decreased (i.e., the standards and targets/deviants became more similar) with increasing performance and vice versa. The 15 target and 15 deviant trials were evenly distributed within consecutive trial sequences of 10 trials. Thus, within 10 consecutive trials, 4 low SF standards, 4 high SF standards, one target, and one deviant occurred in a randomized fashion with the exception that the target and the deviant could not occur as the first or the last trial in a sequence. To enable learning during the practice trials, participants got feedback about the correctness of their decisions. During the actual MEG test phase, participants received no feedback about the correctness of their decisions. They were not informed about how many stimuli were in total or how many targets or deviants would follow. Each MEG run lasted for approximately 8–10 min.

### Experimental protocols

**Figure 2:**
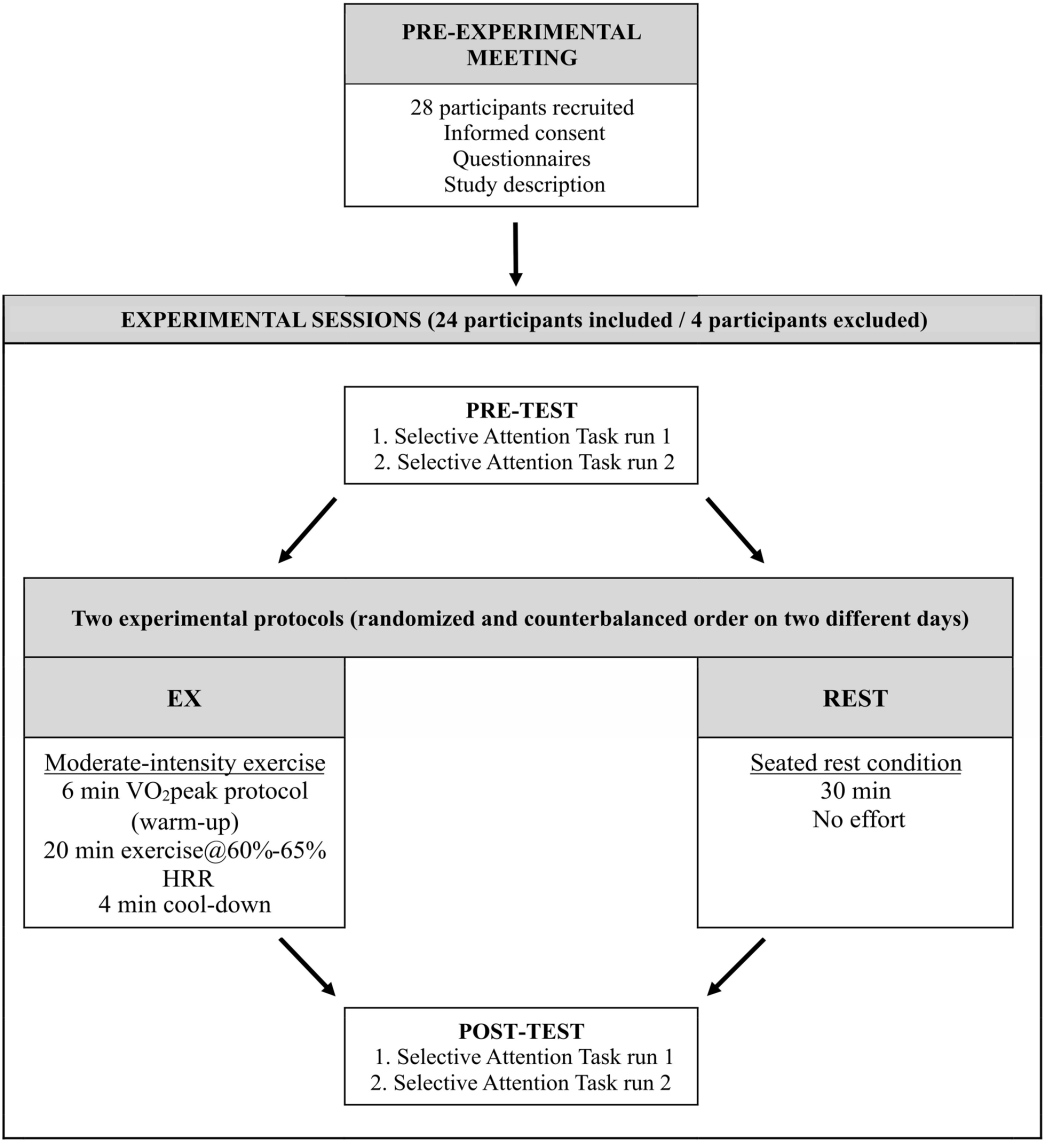
The study diagram.

The present study consisted of two protocols in two separate experimental sessions: the exercise (EX) protocol and the seated rest condition (REST) protocol. Additionally, each participant underwent the VO_2_peak protocol to assess their physical fitness during the EX protocol. The EX and VO_2_peak protocols were administered using a cycle ergometer (Ergoline, Germany). The study overview is presented in Fig. 2.

#### *VO*_*2*_*peak (peak oxygen uptake) protocol*

Peak oxygen uptake (VO_2_peak) was indirectly estimated using the Ǻstrand-Rhyming cycle test to assess the physical fitness of participants. This test assumes a linear relationship between HR and VO_2_peak^39^. All participants performed submaximal effort at constant power for 6 min. The power output was adjusted to the physical capability of each participant so that they would reach the target HR zone of 115–140 bpm at the end of the 6-min test. The mean HR for the last 2 min of the protocol was calculated, and the absolute VO_2_peak value was determined. The pedaling rate during the test was set to 60 rpm. The average load used in the protocol was 87.14 ± 13.19 W.

#### Exercise (EX) and seated rest control (REST) protocols

Parameters for the exercise (EX) protocol were chosen based on a meta-analysis of the effects of acute exercise on cognitive performance^2^. This work has shown that acute aerobic exercise, lasting at least 20 min, is more effective in improving cognition than other forms of exercise (anaerobic, resistance training). This work has also shown that when a cognitive task is performed following a delay of more than 1 min after exercise, more intense exercise results in greater effects than less intense exercise. Therefore, we utilized a protocol of 20 min of aerobic exercise of vigorous intensity. The operationalization of the protocol intensity was based on international guidelines, which consider vigorous intensity as an intensity of 60–89% heart rate reserve (HRR, American College of Sports Medicine, 2018^40^). To make the protocol safer and more pleasant for the participants and more comparable with the majority of studies (which have used moderate-intensity exercise), we aimed to use the exercise intensity closer to the lower range of the vigorous range, namely 60–65% of HRR.

Overall, the utilized exercise protocol (EX) consisted of a 6-min warm-up (indirect VO_2_peak protocol), 20 min of vigorous-intensity continuous exercise, and a 4-min cool-down phase (30 min in total). The workload in the warm-up phase was set according to the VO_2_peak protocol, whereas the workload for the exercise phase was adjusted individually for each participant according to their HRR, which was calculated as the difference between the maximal heart rate (HRmax) and their resting heart rate (HRrest). The maximal heart rate was calculated using the formula HRmax. = 211—(0.64 × age in years)^41^. To determine HRrest, we asked each participant to record their heart rate twice (by palpation) immediately after waking up in the morning for three consecutive days prior to the first testing session. Before taking the measurements, participants were instructed on correctly measuring HRrest. They were asked to count heartbeats within a 60-s period; then, HRrest was calculated as a mean of the six reported measurements (3 days × 2 measurements). During the cool-down, the workload was set to 50% of the average workload used during the exercise phase. Participants were asked to maintain a pedaling rate of 60–70 revolutions per minute during the whole EX protocol. An experimenter adjusted the bike’s resistance on an ongoing basis to meet the participant’s pre-determined HR zones.

During the control condition, namely the seated rest condition (REST), participants were asked to sit in the exercise room and were instructed to relax or read the provided sport-related magazines for 30 min.

## Experimental procedure

The two experimental sessions were conducted at the same time of day on two separate days, 2–22 days apart. The order of the experimental protocols (EX/REST) during experimental sessions was randomized and fully counterbalanced across the final 24 participants. Upon the first visit to the laboratory, participants were provided with the study description, signed informed consent, and were asked to hand in information about their body weight, height, and HRrest. Then, they completed the BSA questionnaire and proceeded with the pre-test MEG session.

Each of the MEG sessions consisted of two runs divided by short breaks. Participants attended a pre-test MEG run and then performed one of the experimental protocols in an adjacent exercise room. During both experimental protocols, participants’ heart rate (HR) was measured using an HR belt chest strap (Polar H10, Finland). In the last 30 s of the EX or REST protocols, participants were asked to rate their perceived exertion during the protocol using the Borg scale (from 6 to 20 points; 6 = no exertion at all; 20 = extremely heavy work)^42^ and provide the score immediately after the protocol ended. Approximately 10 min after the protocol ended, participants underwent the post-test MEG run. One of the measurements was followed by an acquisition of the individual head shape (Polhemus). The overview of the study is provided in Fig. 2.

### Magnetoencephalographic recordings and preprocessing

Continuous MEG data were recorded at a sampling frequency of 600 Hz using a 275-channel whole-head system (Omega 275, CTF, VSM Medtech Ltd., Coquitlam, British Columbia, Canada) with first-order axial superconducting quantum interference device (SQUID) gradiometers (2 cm diameter, 5 cm baseline, 2.2 cm average inter-sensor spacing). Online low-pass hardware filtering of 150 Hz was applied during continuous data acquisition. During the measurements, the participants were wired with three electrocardiographic electrodes that were placed above the right clavicle, on the sternum, and left inferior costal arch to assess movements caused by heart rate and breathing. Participants were placed in an upright sitting position. The head position was stabilized by cotton pads to minimize head movements. Movement data were recorded continuously and monitored via landmark coils attached to the two auditory canals and the nasion.

The preprocessing of MEG data was carried out using the MATLAB-based Electromagnetic Encephalography Software EMEGS^43^ (Version 3.2). Offline MEG data were down-sampled (300 Hz) and filtered with a 0.1-Hz high-pass filter (zero-phase second-order Butterworth) and a 48-Hz low-pass filter (zero-phase fourth-order Butterworth). Trials were split into 800-ms epochs ranging from − 200 to 600 ms relative to stimulus onset and baseline adjusted using the 150 ms before stimuli onset (from − 150 to 0 ms) as the baseline interval. To prevent stimulus offset effects and effects due to preparation for stimulus offset, stimuli were presented for 800 ms and, thus, 200 ms longer than the time interval of interest. Epochs with artifacts were detected and rejected using an established method for statistical control of artifacts in high-density EEG/MEG data^44^. This procedure detects individual channel and global artifacts and replaces artifact-contaminated sensors by spline interpolations while computing signal variance across trials as an estimate of the stability of the averaged waveform. The rejection of artifact-contaminated trials and the interpolation of artifact-contaminated sensors rely on the calculation of statistical parameters for the absolute measured magnetic field amplitudes over time, their standard deviation over time, as well as on the determination of boundaries for each parameter based on their distribution across trials. If the goodness of test topography interpolations based on the residual sensor configuration within a given trial did not reach a priori-defined minimum criterion (k = 0.01; identical for each subject, session, and run), the respective trial was rejected. If more than 30% of the trials in any run of either the pre-test or the post-test runs did not meet this criterion (e.g., due to continuous movement artifacts), the respective participant was rejected from the MEG analysis.

Subsequently, within each individual, epochs of visually evoked magnetic fields (VEMFs) were averaged depending on the time of measurement (pre-test/post-test), experimental task condition (low SF attended/low SF unattended/high SF attended/high SF unattended standard stimuli) and experiment protocol (REST/EX).

On average, 55.2 of the 60 total standard trials for each experimental condition were used for the final average. Underlying neural sources were estimated using the L2-Minimum-Norm-Estimates method (L2-MNE)^45^, which does not rely on a priori specification of the location and/or a number of neural sources^46^ and, thus, is well suited to estimate distributed neural network activation. A spherical shell with evenly distributed 2 (azimuthal and polar direction) × 350 dipoles was used as the source model, and 87% of the individually fitted head radius was used as the source shell radius. Based on the averaged epochs, L2-MNE topographies were calculated for each participant, session, and run and for each experimental condition using a Tikhonov regularization parameter k of 0.1. Topographies of source direction-independent neural activities – the vector length of the estimated source activities at each position – were calculated for each individual participant, condition, and time point.

To avoid statistical artifacts due to potential outliers in any session, run, or experimental condition, participants were excluded if the mean of the standard deviation between experimental conditions across time (N = 2) or if the mean number of trials across experimental conditions (N = 1) differed from the sample median by more than four standard deviations. Finally, the estimated neural activity of attended high and attended low SF and unattended high and unattended low SF, respectively, were averaged within each session as differential modulations by exercise vs. rest for the different low or high spatial frequencies were of no interest.

A 2 × 2x2 ANOVA with the factors time of measurement (pre-test/post-test), experimental task condition (attended/unattended), and an experimental protocol (EX/REST) revealed a significant (F(1,23) = 14.08; *p *< 0.001) but in absolute numbers only slight difference of the number of artifact-free trials in the attended (M = 55.77 trials) vs. unattended (M = 54.52 trials) condition. It is highly unlikely that the difference of 1.25 (~ 2%) trials affected the later-described main effect of attention. Moreover, as event-related fields typically decrease with increasing numbers of averaged trials (i.e., mainly due to phase shifts across trials), the overall stronger neural responses to the attended stimuli (as shown below) could not be explained by the higher number of trials in the attended condition. All other main effects and interactions, and, importantly, all interactions with the experimental factor protocol (EX/REST), were non-significant (all *p *> 0.26).

### Data analysis

Statistical analyses of exercise and behavioral data were performed using the R package and JASP version 0.14.1 (JASP team 2020) software. Statistical analyses of MEG data were performed using the MATLAB-based Electromagnetic Encephalography Software EMEGS^43^ (Version 3.2). The analyses, which aimed to test the main effects of manipulations, were pre-determined before the data collection process based on our research objectives, hypotheses, and the available literature. In case of significant effects, we conducted additional post-hoc tests aimed at in-depth exploration of the obtained effects. Where applicable, post-hoc tests were performed with a Bonferroni correction. Throughout the following description, we provide details on the nature of each test that was conducted.

#### Exercise intervention manipulation

To test the effectiveness of the experimental manipulation, we used pre-determined paired t-tests. Each t-test was calculated separately for the mean heart rate (HR) and the rate of perceived exertion (RPE) measured during both experimental protocols.

#### Behavioral measures

To test the influence of the exercise on behavioral measures, pre-determined 2 × 2x2 ANOVAs were performed for the correctness of the responses to target and non-target stimuli. As the main variable in the study is the brain response to attended/unattended stimuli, we additionally analyzed the responses to these stimulus categories. In both cases, the ANOVA consisted of a 2-level within-subject factor PROTOCOL (EX, REST), a 2-level within-subject factor TIME (pre-test, post-test), and a 2-level within-subject factor TASK (target, non-target or attended, unattended). We were interested in the effects of TASK (as a manipulation check for the task), PROTOCOL*TIME (which reflects the effects of the exercise protocol on behavioral measures), and PROTOCOL*TIME*TASK (which reflects the potentially different effects of exercise on different stimuli types). In case of significant effects, these tests were followed by post-hoc tests. Given the lack of time pressure imposed in the task, we did not analyze reaction times in the main text (see Supplementary Materials for reaction times analyses).

#### Neural measures

Both pre-determined data-driven and post-hoc region-of-interest (ROI) analyses were performed. The data-driven approach was applied to examine the main effect of the task as well as the effects of exercise on attention. The ROI analysis was used post-hoc to additionally test the effects of exercise on attention. We combined data-driven and ROI analysis to provide a more comprehensive and balanced approach. In the case of significant effects of both analyses, further post-hoc tests were utilized.

As for the data-driven analysis, a cluster permutation approach^47^ was applied to the examined effects. This approach takes the problem of multiple comparisons for the analysis of distributed sources into account, does not rely on a normal distribution of the estimated neural data, and does not rely on a priori specification of regions of interest or time intervals of interest. More specifically, the statistical value of each time point and dipole was tested for significance with α-levels of *p-sensor* = 0.05 or *p-sensor* = − 0.05 (sensor-level criterion). The so-called cluster mass is calculated by the spatiotemporal integral of the statistical value, the spatial extent, and the temporal extent. Afterward, the actual cluster mass was tested against 1,000 permuted random drawings, which were drawn from the original data set as well. The distribution of the permutations was employed to determine the cluster criterion. If the actual cluster mass exceeded the critical cluster mass of *p-cluster* = 0.05 (i.e., greater than 95% of the heaviest sensor-level significant clusters identified in each of the 1,000 permutations; cluster-level criterion), the effect was classified as significant. To avoid false temporal precision, cluster onset, and offset were rounded to the nearest 10 ms. For visualization purposes, L2-MNE topographies were projected on standard 3D brain models.

Using a data-driven approach, we first conducted a paradigm check to determine whether we could replicate the expected baseline effects of increased neuronal activity for attended vs. unattended stimuli (i.e., the main effect of TASK). To test this main effect of TASK, we merged both baseline measures (pre-test) runs of the EX and REST sessions and compared the attended and unattended conditions by paired t-tests performed for each time point and test dipole.

We conducted two analyses to investigate the effects of exercise on attentional processing: a pre-determined data-driven cluster permutation approach and a post-hoc ROI analysis. The ROI analysis focused on spatiotemporal regions that exhibited the strongest effects of attention (i.e., the main effect of the task) and was performed to validate the attentional nature of the exercise effects based on a priori expectations. In the data-driven approach, we again utilized the cluster permutation method. This analysis aimed to reveal a broad overview of brain activity across different regions and times independent of a priori-defined time intervals and regions of interest. To this end, we calculated the change in the difference between attended and unattended stimulus processing by subtracting the post-test run difference from the pre-test run difference. We then compared the EX and REST protocols using paired t-tests at each time point and test dipole.

Both ROI and data-driven analyses that concerned the exercise effects were followed by post-hoc tests (ANOVAs) that tested for the interactions between relevant factors. In each case, ANOVA consisted of a 2-level within-subject factor PROTOCOL (EX, REST), a 2-level within-subject factor TIME (pre-test, post-test), and a 2-level within-subject factor TASK (attended, unattended).

Note that our procedure, optimized for analyzing brain responses to attended and unattended standard stimuli, provided an insufficient number of trials available for reliable analysis of brain responses to target and deviant stimuli. Furthermore, analyses of brain responses to targets would be further complicated by the changing physical properties of this type of stimuli throughout the experiment.

## Results

### Exercise intervention (manipulation check)

The mean heart rate (HR) during the exercise (EX) protocol (*M* = 145.67; *SD* = 5.73) was significantly higher (*t* = 41.24, *p* < 0.001) than during the seated resting (REST) protocol (*M* = 65.83; *SD* = 8.93). Convergently, the rate of perceived exertion (RPE) during the EX protocol (*M* = 12.92; *SD* = 1.16) was significantly higher (*t* = 25.38, *p* < 0.001) than during the REST protocol (*M* = 6.29; *SD* = 0.70). The detailed characteristics of the workloads during the experimental protocols are presented in Table 2.

**Table 2:** Characteristics of experimental protocols (M ± SD).

	EX	REST
RPE (Borg scale)	12.92 ± 1.16	6.29 ± 0.70
RPE (verbal anchor)	“Somewhat hard”	“No exertion at all”
Mean HR (bpm)	145.67 ± 5.73	65.83 ± 8.93
% HRmax	73.91 ± 3.50	33.39 ± 4.35
% HRR	62.45 ± 5.10	n / a
Mean power (watt)	117.53 ± 29.81	n / a

*RPE* Rate of perceived exertion, *HR* Heart rate, *HRR* heart rate reserve, *HRmax* Maximum heart rate, *EX* Vigorous exercise at bike ergometer, *REST* Seated rest condition.

### Behavioral measures

The mean correctness for targets, non-targets, attended and unattended stimuli that were assessed before (pre-test) and after (post-test) the experimental protocols are shown separately for each PROTOCOL (EX and REST) in Table 3 (see Supplementary Materials for RT analyses ).

**Table 3:** Behavioral results – accuracy (%).

	Pre-test (M ± SD)	Post-test (M ± SD)
EX protocol		
Targets	93.47 ± 3.87	93.47 ± 2.67
Non-targeTS	99.26 ± 1.49	99.31 ± 2.67
Attended	98.33 ± 3.34	98.44 ± 3.03
Unattended	100 ± 0	99. 97 ± 0.17
REST protocol		
Targets	94.03 ± 2.78	93.61 ± 2.77
Non-targets	98.95 ± 1.92	99.60 ± 0.86
Attended	97.81 ± 4.16	99.13 ± 1.94
Unattended	99.82 ± 0.42	100 ± 0

*EX* Vigorous-intensity exercise, *REST* Seated rest condition.

#### Targets/non-targets

The analyses revealed a significant effect of TASK [*F*(1,23) = 178.89, *p* < 0.001, *η2* = 0.87]. Post-hoc test showed that the average correctness for targets [*M* = 93.65; *SD* = 2.04] was significantly lower [*p* < 0.001] than for the non-targets [*M* = 99.28; *SD* = 0.93]. The PROTOCOL*TIME and PROTOCOL*TIME*TASK effects were non-significant.

#### Attended/unattended

The analyses revealed a significant effect of TASK [*F*(1,23) = 13.27, *p* < 0.001, *η2* = 0.37]. Post-hoc test showed that the average correctness for attended stimuli [*M* = 98.43; *SD* = 2.06] was significantly lower [*p* < 0.001] than for the unattended stimuli [*M* = 99.95; *SD* = 0.11]. The PROTOCOL*TIME and PROTOCOL*TIME*TASK effects were non-significant.

### MEG data

#### The main effect of TASK

**Figure 3:**
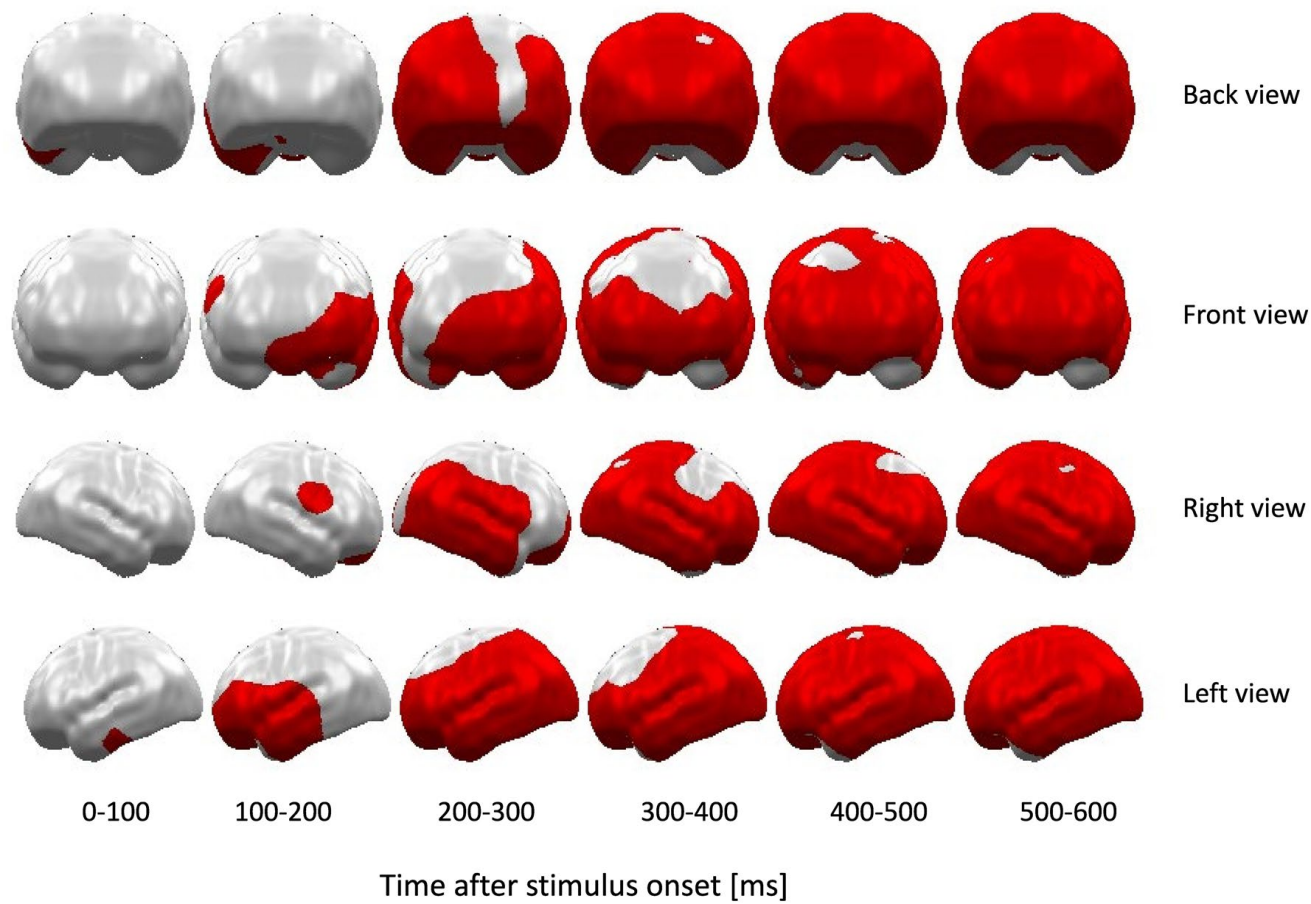
Time course and topographies of the spatiotemporal cluster showing a significant main effect of TASK (attended vs. unattended stimuli) at both pre-test runs, i.e., before the experimental EX or REST protocols. As expected, attended stimuli evoked significantly stronger neural activation in a widely distributed spatiotemporal cluster starting around 90 ms after stimulus onset. There were no significant clusters with relatively stronger neural activation for unattended stimuli.

As expected, the cluster permutation-based paired t-test of TASK effects (attended vs. unattended) of both pre-test (i.e., baseline) sessions (i.e., unaffected by the subsequent experimental exercise [EX] or rest [REST] protocols) revealed a very strong, sustained, and widely distributed effect of TASK (p-*cluster* < 0.001) with overall stronger neural activity in response to attended compared to unattended stimuli (see Fig. 3). This spatio-temporal cluster started to become significant around 90 ms after stimulus onset in the left superior temporal areas and rapidly expanded in time and space. There were no significant clusters with relatively stronger neural activation for unattended stimuli (p-*cluster* > 0.9).

#### ROI-based analysis of the TASK effect to test exercise effects

**Figure 4:**
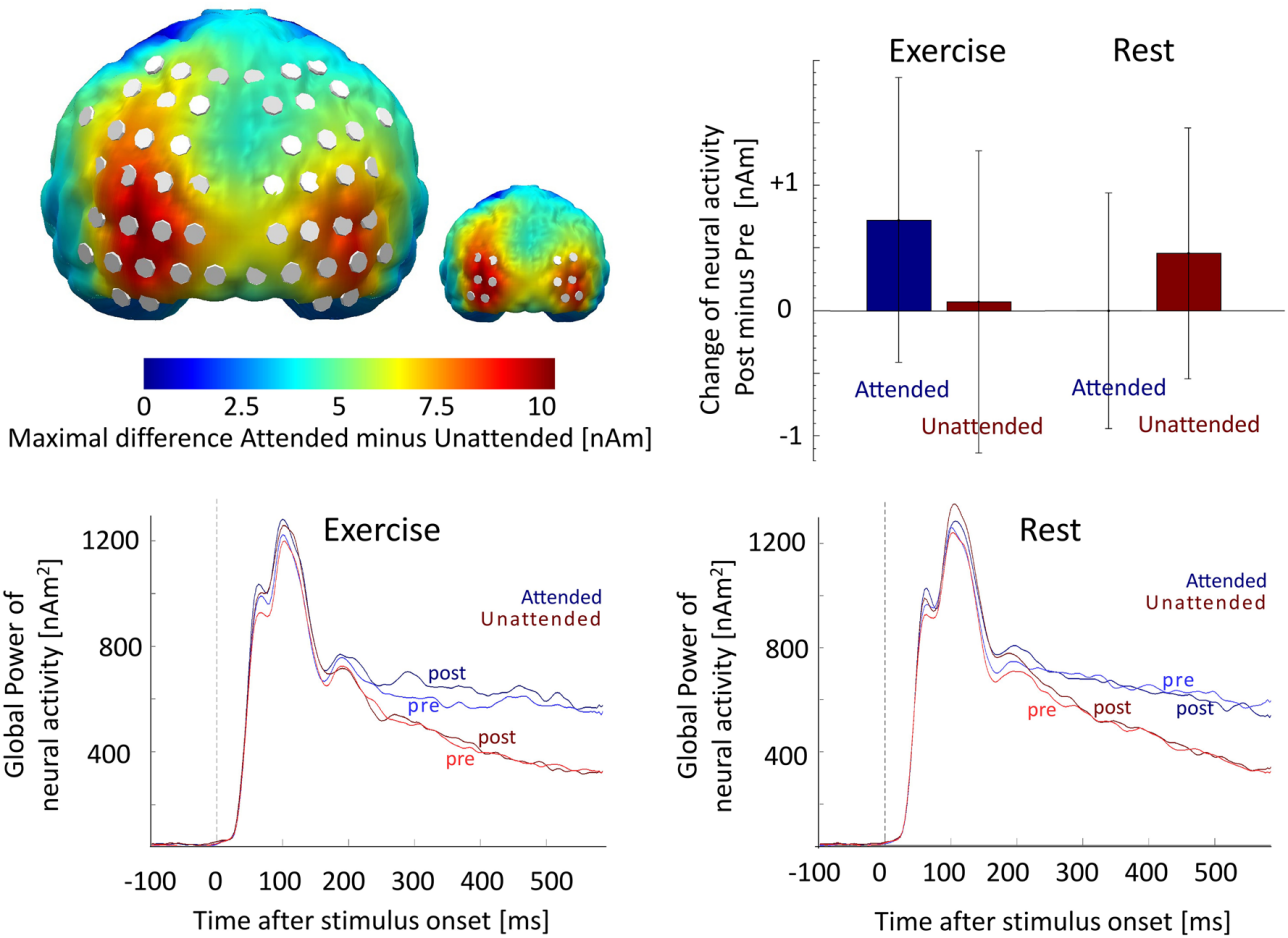
Left Top: Topography of strongest neural difference activities between attended and unattended stimuli in both baseline runs (i.e., before exercise and before rest). An occipito-parietal region of interest was defined by estimated neural sources showing at least 50% of the maximal difference. Effects remained qualitatively identical when regions of interest with 60%, 70%, or 80% of the maximal difference were chosen (small topography shows 80% region of interest) and when taking into consideration the overall difference between attended and unattended stimuli (i.e., before and after exercise and rest). Bottom: Time courses of the global power of estimated neural activities within the bilateral occipito-parietal regions of interest as defined above (difference > 50%) for the Exercise (left) and Rest (right) conditions. Based on a sliding window analysis of neural activity within these regions of interest, attended stimuli (blueish colors) evoked stronger neural activity than unattended stimuli (reddish colors) before (bright colors) and after (dark colors) the protocols, starting around 90 ms after stimulus onset in the left hemisphere. Right Top: Relative change of neural activity from pre- to post-protocol between 90 and 600 ms in the 50% region of interest. While the pre-test (“normal”) relation of neural activity (i.e., attended > unattended) relatively increased after exercise (i.e., a relatively stronger increase of attended vs. unattended), this relation decreased after rest. Error bars denote 95% confidence intervals.

A post-hoc analysis testing the modulation of neural activity in the above spatiotemporal cluster (Fig. 3) by the experimental protocols, i.e., 2 × 2x2 ANOVA of PROTOCOL (exercise/rest) * TIME (pre/post) * TASK (attended/unattended) did not reveal a significant 3-way interaction. However, a post-hoc analysis focusing on the occipito-parietal regions showing the strongest neural difference activities between attended and unattended stimuli in both baseline runs that were at least 50% (5.44 nAm) of the maximal difference (10.88 nAm) (see Fig. 4) revealed significant modulation of PROTOCOL*TIME*TASK (*F*(1,23) = 5.630*, p* = 0.026, *η*^*2*^ = 0.197). This effect was almost identical when taking into account regions of interest based on the overall differences of attended vs. unattended stimuli (i.e., before and after exercise and rest) (*p *= 0.025). This effect was also qualitatively identical when taking into account regions with 60%, 70%, or 80% of the strongest differences in neural activity between attended and unattended stimuli (0.026 < *p *< 0.032). Importantly, while in the occipito-parietal areas the EX compared to the REST protocol had no effect on attention in the pre-test (PROTOCOL*TASK: *F* < 1), in the post-test the EX compared to the REST protocol relatively increased the neural difference activation between attended and unattended stimuli (*F*(1,23) = 4.790, *p* = 0.039, *η*^*2*^ = 0.172). Additionally, while after exercise the pre-test (“normal”) relation of stronger neural activity for attended compared to unattended stimuli relatively increased by trend (post minus pre; attended vs. unattended: *t*(23) = 1.736; *p* < 0.066, *d* = 0.354), after rest this relation decreased, though not significantly (*t*(23) =  − 1.131; *p* = 0.270, *d* = − 0.231). A post-hoc sliding window PROTOCOL*TIME*TASK analysis (50 ms windows) with a specific focus on effect latency revealed that the 3-way interaction showed significance (*p* < 0.05) between 250 and 320 ms after stimulus onset in the bilateral clusters, but it showed significance already between 130 and 180 ms after onset in the left hemispheric cluster. A post-hoc analysis revealed that the relatively increased attention effects after exercise compared to after rest were comparable in the left and right hemispheres (PROTOCOL*TIME*TASK*HEMISPHERE: F < 1).

#### Data-driven effects of exercise on selective attention

**Figure 5:**
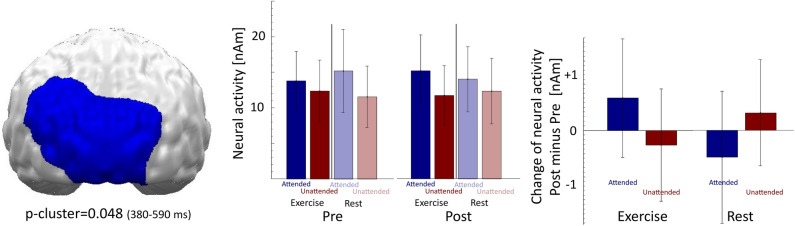
left: Topography (back view) of the spatiotemporal cluster showing a significant 3-way PROTOCOL*TIME*TASK interaction at bilateral occipito-parietal cortex regions between 380 and 590 ms. Center: In this cluster, independent of TIME (pre/post) or PROTOCOL (exercise/rest), attended stimuli evoked stronger neural activity than unattended stimuli (i.e., the main effect of TASK). Right: While the pre-test (“normal”) relation of neural activity (i.e., attended > unattended) became relatively strengthened after exercise (i.e., a relatively stronger increase of attended compared to unattended), this relation decreased after rest. Error bars denote 95% confidence intervals.

The paired t-test cluster-based permutation analysis (*attended (post minus pre) minus unattended (post minus pre) for EX vs. REST*) of estimated neural activity revealed a significant spatiotemporal cluster (p-*cluster* = 0.048) between 380 and 590 ms within occipito-temporo-parietal cortex regions (see Fig. 5 left panel).

As expected, the post-hoc 3-way PROTOCOL (exercise/rest) * TIME (pre/post) * TASK (attended/unattended) ANOVA testing neural activity within this cluster confirmed the strong main effect of TASK (*F*(1,23) = 31.120, *p* < 0.001, *η*^*2*^ = 0.575) as well as the tested 3-way interaction (*F*(1,23) = 31.442, *p* < 0.001, *η*^*2*^ = 0.578). Separate post-hoc 2-way PROTOCOL*TASK ANOVAs for the pre-test and post-test runs again confirmed highly significant effects of TASK in both pre-test (*F*(1,23) = 33.019, *p* < 0.001, *η*^*2*^ = 0.589) and post-test (*F*(1,23) = 22.702*, p* < 0.001, *η*^*2*^ = 0.497). These attention effects were modulated by PROTOCOL in the post-test (*F*(1,23) = 10.243, *p* = 0.004, *η*^*2*^ = 0.308) but also in the pre-test (*F*(1,23) = 20.900, *p* < 0.001, *η*^*2*^ = 0.476). While the pre-test (“normal”) relation of neural activity (i.e., attended > unattended) within this region became significantly strengthened after exercise (post minus pre; attended vs. unattended: *t*(23) = 4.750*; p* < 0.001, *d* = 0.970), this relation significantly decreased after rest (*t*(23) =  − 3.310; *p* = 0.0031, *d* = 0.676). Post-hoc t-tests revealed a significant increase of neutral activity for attended stimuli after exercise (*t*(23) = 2.677; *p* = 0.013, *d* = 0.546) and a trendwise decrease for attended stimuli after rest (*t*(23) = − 2.005; *p* = 0.057, *d* = − 0.409) while processing of unattended stimuli did not differ between the pre- and post-measure in the exercise condition (*t*(23) = − 1.286; *p* = 0.211, *d* = − 0.263) or in the rest condition (*t*(23) = 1.713; *p* = 0.124, *d* = 0.326). A post-hoc sliding window PROTOCOL*TIME*TASK analysis (50 ms windows) with a specific focus on effect latency revealed that this 3-way interaction showed significance (*p* < 0.05) in this region between 240 and 590 ms after stimulus onset.

Thus, both post-hoc ANOVA comparisons of the strongest main effects of TASK (attended > unattended) after exercise and rest (ROI analysis), as well as data-driven permutation tests for modulations of attention effects by the experiential protocols, revealed that exercise vs. rest modulated neural responses to attended and unattended stimuli within bilateral occipito-parietal cortex regions. These effects were not lateralized and were strongest at mid-latency and late processing stages, although the earliest effects became visible around 130 ms after stimulus onset.

## Discussion

We investigated the effect of acute aerobic exercise (hereafter, “exercise”) on neural markers of selective attention, which was maximally isolated from higher cognitive processes such as decision-making or responding. We found that exercise, relative to a seated rest condition (control condition), facilitated the neural responses to attended vs. unattended stimuli. As compared with previous evidence, our study provides a novel, more direct, and more robust evidence to support the claim that exercise benefits efficient attentional resource deployment during cognitive processing.

To assess neural markers of selective attention, we measured MEG responses during a modified spatial frequency attention task^28^. Participants were asked to identify infrequent target stimuli among standard stimuli of attended and unattended frequency and were allowed to respond after stimuli offset. We focused on the difference in neural responses to attended and unattended stimuli during stimuli perception. In line with the general assumption about selective attention and in line with previous neuroimaging evidence, in all of the task runs, attended vs. unattended stimuli evoked strong and widely distributed neural difference activations (attended > unattended) that started around 90 ms after stimulus onset at superior temporal cortex regions, dovetailing in space and time with results of the first multi-modal neuroimaging study on selective attention^48^. The most significant differences between attended and unattended stimuli occurred in bilateral occipito-parietal and superior temporal cortex regions, again consistent with previous studies^28,48–50^. Overall, the spatiotemporal effects of TASK (attended vs. unattended stimuli) are consistent with previous findings.

The task was completed before (pre-test) and after (post-test) exercise and rest protocols, allowing us to understand the effects of exercise on selective attention. First, we utilized a region of interest (ROI) analysis to verify whether the effects of TASK (described above) were modulated by the experimental protocols (PROTOCOL) and measurement time (TIME). Second, to directly test our hypothesis, we ran data-driven, neural activity cluster analyses that tested specifically for the interactive effects of PROTOCOL*TIME*TASK factors. As described above, both analyses suggest that exercise (compared to rest) facilitates the neural differentiation of attended vs. unattended stimuli.

Regarding the first (ROI) analysis, we verified whether TASK effects were modulated by exercise (specifically, PROTOCOL*TIME interaction). Although exercise did not modulate the whole activity of the cluster that showed a main effect of TASK, exercise modulated the activity in bilateral occipito-parietal cortex regions where the effects of TASK were maximal. While the difference in neural activity in the occipito-parietal cluster in response to attended vs. unattended stimuli (TASK effect) did not differ between exercise and rest during the pre-test, during post-test this difference was more pronounced after exercise. Exercise thus increased the processing of attended relative to unattended stimuli compared to the rest protocol. The localization of this effect is consistent with our assumptions and suggests that exercise modulates the activity of regions that play a crucial role in the attention to visual stimuli^28,48–51^. Post-hoc tests focused on the timing of these effects suggested a relatively early onset of the effect, around 130 ms for left hemispheric and slightly later onset for right hemispheric effects, indicating that neural activations of attention occurred in the time interval of the so-called selection negativity interval (SN, 140–180 ms)^51^ but, presumably, not in the preceding P1 time interval^48^. The timing of the effect indicates that this modulation might start quite early in cognitive processing, distinctly preceding more complex higher-order processes such as decision-making or responding. Although the studies so far have primarily indicated modulations of later components related to attention (P3b), our results suggest that exercise may also affect earlier attentional markers, as reported when ERPs were measured during exercise ^25,26^.

The second analysis directly focused on the interactional effect of PROTOCOL*TIME*TASK on neural activity and identified a spatiotemporal cluster between 380–590 ms at occipito-temporo-parietal cortex regions. The difference in neural activity in this cluster in response to attended vs. unattended stimuli increased after exercise (compared to pre-test), whereas this relation decreased after rest. Again, the localization of the cluster suggested that certain regions are responsible for the processing of perceptual stimuli. The timing of this effect suggests that modulation of attention occurs at a later stage of stimuli evaluation and is in line with the timeline of exercise-induced modulation of ERP responses in cognitive tasks following exercise (marked by the P3b component)^16^. As such, our source localization analysis provides more robust and direct evidence to support the hypothesis that exercise-induced neural effects in the observed time window might be attentional in nature. We base this supposition on the location of the effect as well as the attention-demanding nature of the task we used. However, we cannot rule out that during these relatively late processes of perceptual stimuli processing, processes other than attentional ones may (at least in part) have been responsible for the observed effect.

Overall, both analyses suggest that exercise influences neural activation related to selective attention. Although our first analysis indicates that some modulations of attention may begin in the early perceptual stages of stimulus processing, the main effect of exercise is evident during the later cognitive stages. An additional analysis performed separately on attended and unattended stimuli suggests that exercise primarily increases neural activation related to the processing of attended stimuli rather than decreasing neural activation related to the processing of unattended stimuli. This finding suggests that exercise may enhance attentional resources, allowing for increased allocation of cognitive resources to processing stimuli relevant to the task at hand.

The exercise-induced changes in neural activations were not accompanied by changes in task performance. Given the very high overall task accuracy in all runs, the task might have been too easy to reveal exercise effects. This ceiling effect could have resulted from optimizing the experimental procedure to observe neural effects. For example, the lack of time pressure in responding might have helped participants respond more accurately at the cost of reaction times (which were not assessed in our study). A meta-analysis examining acute exercise's effects on accuracy and reaction times revealed that reaction times significantly improved after exercise, with minimal or no effect on accuracy^52^. Moreover, exercise-induced changes in behavioral measures do not always accompany effects in neural measures. For example, according to a meta-analysis on the effect of exercise on the P3b component, while 19 of 23 studies showed positive effects of exercise on the P3b component, 4 of these studies could not show positive behavioral effects, suggesting that neural measures might be more sensitive to detecting exercise-induced changes in cognition^16^. This seems to be especially true when the task is too easy and only accuracy is measured.

We, nevertheless, cannot clearly state that the observed patterns of neural activations served better task performance. Considering the ceiling effect in behavioral data, it cannot be stated whether a more efficient allocation of neural resources was facilitative and potentially allowed for better task performance (if the task had been more difficult, longer, or more complex) or whether exercise was detrimental to attentional capacity so that greater neural efficiency was needed to maintain the high level of the performance. Given the predominant pattern of results obtained in other studies (indicating positive effects of exercise on cognition), it seems more likely that the observed neural changes could be facilitative. Moreover, it seems reasonable to argue that more neural activation within regions necessary for the processing of task-relevant stimuli might benefit subsequent decision-making and responses. As such, the obtained pattern of results could support the general improvement hypothesis, stating that the benefits of exercise are observed regardless of the specific tasks used or the specific task conditions analyzed. However, to unequivocally confirm these suppositions, future research should use more complex tasks to understand whether the neural changes can indeed be translated into better task performance.

Several mechanisms, including neurogenesis, neural plasticity, and changes in neurotransmission or cerebral metabolism, have been proposed to account for the acute effects of exercise on cognition^53^. One of the suggested mechanisms assumes that after exercise, the locus coeruleus, a brainstem nucleus that integrates signals about arousal from various brain systems, releases more norepinephrine. Norepinephrine is known to modulate cortical arousal that serves for improved attention and vigilance. Under arousal, salient stimuli stand out even more than they would otherwise, while less salient stimuli are more likely to be ignored^54–56^ These suggested effects of increased norepinephrine resemble the neural effects observed in the study at hand: increased neural activation in response to attended stimuli vs. decreased response to unattended stimuli. As such, the exercise-induced changes in norepinephrine release likely and at least in part accounted for the observed effects. Future studies should assess whether an exercise-induced change in norepinephrine levels is indeed associated with exercise-induced changes in neural activation related to selective attention.

Despite these new insights in exercise-driven modulations of selective attention, there are limitations to consider. As already discussed, behavioral effects were not observed, significantly limiting the possibility of unequivocal data interpretation. Another limitation of our study is that we used exercise of only vigorous intensity. The obtained results could be different when considering lower or higher exercise intensity. Furthermore, for the analysis of the interactional effect of PROTOCOL*TIME*TASK, we observed some differences already at the pre-test, when rest and exercise protocols were compared. Although participants were not informed about the order of the sessions, it is very likely that after participating in the first session (e.g., rest), they may have been able to predict the protocol of the second session (i.e., exercise). This could somehow have affected their expectation prior to the manipulation. On the other hand, the region and time interval of interest-driven analysis on the most potent effects of TASK showed no difference in the pre-test, indicating that the observed pre-test difference could have been related to natural, day-to-day variability in measurements.

Overall, our findings contribute to the growing body of evidence that exercise has positive effects on cognitive function, particularly on attention. We showed that exercise might benefit more efficient allocation of neural resources related to selective attention. After exercise, more neural resources are deployed for attended stimuli. Thanks to a carefully designed task and advanced source space analysis, our study provides a novel, more robust, and more direct evidence than previous evidence on exercise's effect on attention. By clarifying the mechanisms by which exercise affects neural processing, our study may inform the development of more targeted interventions for cognitive enhancement through exercise. Although it can be speculated that the observed changes in selective attention might, at least in part, explain the general improvements in performance observed across a variety of cognitive tasks, the lack of behavioral effects in this study limits such an interpretation and warrants further investigations. Nevertheless, at the neural level, acute aerobic exercise seems to affect attentional resources related to the processing of attended vs. unattended stimuli.

## Supplementary Information

Supplementary Information.

## Data Availability

All current study data are available from the corresponding author on request.

## References

[1] Ludyga S, Gerber M, Brand S, Holsboer-Trachsler E, Pühse U (2016). Acute effects of moderate aerobic exercise on specific aspects of executive function in different age and fitness groups: A meta-analysis. Psychophysiology.

[2] Chang YK, Labban JD, Gapin JI, Etnier JL (2012). The effects of acute exercise on cognitive performance: A meta-analysis. Brain Res..

[3] Hillman, C. H., Kamijo, K. & Pontifex, M. B. The Relation of ERP Indices of Exercise to Brain Health and Cognition. in *Functional Neuroimaging in Exercise and Sport Sciences* (eds. Boecker, H., Hillman, C. H., Scheef, L. & Strüder, H. K.) 419–446 (Springer, 2012). doi:10.1007/978-1-4614-3293-7_18.

[4] Botvinick MM, Braver TS, Barch DM, Carter CS, Cohen JD (2001). Conflict monitoring and cognitive control. Psychol Rev.

[5] Lambourne K, Tomporowski P (2010). The effect of exercise-induced arousal on cognitive task performance: A meta-regression analysis. Brain Res..

[6] Zhou, F. & Qin, C. Acute moderate-intensity exercise generally enhances attentional resources related to perceptual processing. *Fron. Psychol.* **10**, (2019). 10.3389/fpsyg.2019.02547 PMC6856792 31781010

[7] Ludyga S, Gerber M, Pühse U, Looser VN, Kamijo K (2020). Systematic review and meta-analysis investigating moderators of long-term effects of exercise on cognition in healthy individuals. Nat. Hum. Behav..

[8] Schneider W, Shiffrin RM (1977). Controlled and automatic human information processing: I. Detection, search, and attention. Psychol. Rev..

[9] Neumann, O. Automatic Processing: A Review of Recent Findings and a Plea for an Old Theory. in *Cognition and Motor Processes* (eds. Prinz, W. & Sanders, A. F.) 255–293 (Springer, 1984). doi:10.1007/978-3-642-69382-3_17.

[10] Ligeza TS, Maciejczyk M, Kałamała P, Szygula Z, Wyczesany M (2018). Moderate-intensity exercise boosts the N2 neural inhibition marker: A randomized and counterbalanced ERP study with precisely controlled exercise intensity. Biol. Psychol..

[11] Sibley BA, Etnier JL, Le Masurier GC (2006). Effects of an acute bout of exercise on cognitive aspects of stroop performance. J. Sport Exerc. Psychol..

[12] Condello G (2017). Steps to health in cognitive aging: Effects of physical activity on spatial attention and executive control in the elderly. Front. Hum. Neurosci..

[13] Huertas F, Blasco E, Moratal C, Lupiañez J (2019). Caffeine intake modulates the functioning of the attentional networks depending on consumption habits and acute exercise demands. Sci. Rep..

[14] Luck SJ, Kappenman ES (2013). The Oxford Handbook of Event-Related Potential Components.

[15] Ritter W, Vaughan HG (1969). Averaged evoked responses in vigilance and discrimination: A reassessment. Science.

[16] Kao S-C (2020). A systematic review of physical activity and cardiorespiratory fitness on P3b. Psychophysiology.

[17] Polich J (2007). Updating P300: An integrative theory of P3a and P3b. Clin. Neurophysiol..

[18] Hillman CH, Buck SM, Themanson JR, Pontifex MB, Castelli DM (2009). Aerobic fitness and cognitive development: Event-related brain potential and task performance indices of executive control in preadolescent children. Dev. Psychol..

[19] Hillman CH, Kamijo K, Scudder M (2011). A review of chronic and acute physical activity participation on neuroelectric measures of brain health and cognition during childhood. Prev. Med..

[20] Kamijo K, Nishihira Y, Higashiura T, Kuroiwa K (2007). The interactive effect of exercise intensity and task difficulty on human cognitive processing. Int. J. Psychophysiol..

[21] Chu C-H, Alderman BL, Wei G-X, Chang Y-K (2015). Effects of acute aerobic exercise on motor response inhibition: An ERP study using the stop-signal task. J. Sport Health Sci..

[22] Wu C-H (2019). Effects of acute aerobic and resistance exercise on executive function: An ERP study. J. Sci. Med. Sport.

[23] Chang Y-K (2017). Acute exercise has a general facilitative effect on cognitive function: A combined ERP temporal dynamics and BDNF study. Psychophysiology.

[24] Themanson JR, Hillman CH (2006). Cardiorespiratory fitness and acute aerobic exercise effects on neuroelectric and behavioral measures of action monitoring. Neuroscience.

[25] Pontifex MB, Hillman CH (2007). Neuroelectric and behavioral indices of interference control during acute cycling. Clin. Neurophysiol..

[26] Bullock T, Cecotti H, Giesbrecht B (2015). Multiple stages of information processing are modulated during acute bouts of exercise. Neuroscience.

[27] Verleger R (2020). Effects of relevance and response frequency on P3b amplitudes: Review of findings and comparison of hypotheses about the process reflected by P3b. Psychophysiology.

[28] Mart’nez A, Di Russo F, Anllo-Vento L, Hillyard SA (2001). Electrophysiological analysis of cortical mechanisms of selective attention to high and low spatial frequencies. Clin. Neurophysiol..

[29] Singh SP (2014). Magnetoencephalography: Basic principles. Ann. Indian Acad. Neurol..

[30] Gandhi SP, Heeger DJ, Boynton GM (1999). Spatial attention affects brain activity in human primary visual cortex. Proc. Natl. Acad. Sci. U. S. A..

[31] Hopfinger JB, West VM (2006). Interactions between endogenous and exogenous attention on cortical visual processing. Neuroimage.

[32] Drollette ES, Shishido T, Pontifex MB, Hillman CH (2012). Maintenance of cognitive control during and after walking in preadolescent children. Med. Sci. Sports Exerc..

[33] Pontifex MB, Parks AC, Henning DA, Kamijo K (2015). Single bouts of exercise selectively sustain attentional processes. Psychophysiology.

[34] Kao S-C (2022). Acute effects of aerobic exercise on conflict suppression, response inhibition, and processing efficiency underlying inhibitory control processes: An ERP and SFT study. Psychophysiology.

[35] Ligeza TS, Maciejczyk M, Wyczesany M, Junghofer M (2023). The effects of a single aerobic exercise session on mood and neural emotional reactivity in depressed and healthy young adults: A late positive potential study. Psychophysiology.

[36] Ligeza TS (2020). Acute aerobic exercise enhances pleasant compared to unpleasant visual scene processing. Brain Cogn..

[37] Fuchs R (2015). Messung der Bewegungs- und Sportaktivität mit dem BSA-Fragebogen : Eine methodische Zwischenbilanz. Zeitschrift für Gesundheitspsychologie.

[38] Brainard DH (1997). The psychophysics toolbox. Spat. Vis..

[39] Astrand PO, Ryhming I (1954). A nomogram for calculation of aerobic capacity (physical fitness) from pulse rate during sub-maximal work. J. Appl. Physiol..

[40] American College of Sports Medicine, Riebe, D., Ehrman, J. K., Liguori, G. & Magal, M. *ACSM’s guidelines for exercise testing and prescription*. (2018). 10.1249/JSR.0b013e31829a68cf 23851406

[41] Nes BM, Janszky I, Wisløff U, Støylen A, Karlsen T (2013). Age-predicted maximal heart rate in healthy subjects: The HUNT fitness study. Scand. J. Med. Sci. Sports.

[42] Borg GA (1982). Psychophysical bases of perceived exertion. Med. Sci. Sports Exerc..

[43] Peyk P, De Cesarei A, Junghöfer M (2011). ElectroMagnetoEncephalography software: Overview and integration with other EEG/MEG toolboxes. Comput. Intell. Neurosci..

[44] Junghöfer M, Elbert T, Tucker DM, Rockstroh B (2000). Statistical control of artifacts in dense array EEG/MEG studies. Psychophysiology.

[45] Hämäläinen MS, Ilmoniemi RJ (1994). Interpreting magnetic fields of the brain: Minimum norm estimates. Med. Biol. Eng. Comput..

[46] Hauk O (2004). Keep it simple: A case for using classical minimum norm estimation in the analysis of EEG and MEG data. Neuroimage.

[47] Maris E, Oostenveld R (2007). Nonparametric statistical testing of EEG- and MEG-data. J. Neurosci. Methods.

[48] Heinze HJ (1994). Combined spatial and temporal imaging of brain activity during visual selective attention in humans. Nature.

[49] Corbetta M, Miezin FM, Dobmeyer S, Shulman GL, Petersen SE (1991). Selective and divided attention during visual discriminations of shape, color, and speed: Functional anatomy by positron emission tomography. J Neurosci.

[50] Mangun GR, Hopfinger JB, Kussmaul CL, Fletcher EM, Heinze H-J (1997). Covariations in ERP and PET measures of spatial selective attention in human extrastriate visual cortex. Hum. Brain Mapp..

[51] Hillyard SA, Anllo-Vento L (1998). Event-related brain potentials in the study of visual selective attention. Proc. Natl. Acad. Sci..

[52] McMorris T, Hale BJ (2012). Differential effects of differing intensities of acute exercise on speed and accuracy of cognition: A meta-analytical investigation. Brain Cogn..

[53] McMorris, T., Turner, A., Hale, B. J. & Sproule, J. Beyond the catecholamines hypothesis for an acute exercise–cognition interaction: A neurochemical perspective. in *Exercise-cognition interaction: Neuroscience perspectives* 65–103 (Elsevier Academic Press, 2016). doi:10.1016/B978-0-12-800778-5.00004-9.

[54] Kinomura S, Larsson J, Gulyás B, Roland PE (1996). Activation by attention of the human reticular formation and thalamic intralaminar nuclei. Science.

[55] Lee, T.-H., Itti, L. & Mather, M. Evidence for Arousal-Biased Competition in Perceptual Learning. *Front. Psychol.* **3**, (2012). 10.3389/fpsyg.2012.00241 PMC3400437 22833729

[56] Sutherland MR, Mather M (2012). Negative arousal amplifies the effects of saliency in short-term memory. Emotion.

