# Supplementary material for: Acute aerobic exercise benefits allocation of neural resources related to selective attention
Source: Sci Rep. 2023 May 27;13:8624. doi: 10.1038/s41598-023-35534-5 (PMC10220342; doi:10.1038/s41598-023-35534-5)
Supplement: Supplementary file 1 — Supplementary Information. [file 41598_2023_35534_MOESM1_ESM.docx]

## REACTION TIMES (RT) ANALYSIS

## Data analysis

Statistical analyses of reaction times (RT) data were performed using the R package and JASP version 0.14.1 (JASP team 2020) software. The mean RT of correct trials was calculated within and across the task conditions. Trials with RT that were three standard deviations above or below the condition mean for a given participant were excluded (M = 0.86% of trials).

To test the influence of the exercise on RT measures, 2x2x2 ANOVAs were performed for the RT of the responses to target/non-target stimuli, as well as to attended/unattended stimuli. In both cases, the ANOVA consisted of a 2-level within-subject factor PROTOCOL (EX, REST), a 2-level within-subject factor TIME (pre-test, post-test), and a 2-level within-subject factor TASK (target, non-target or attended, unattended. Where applicable, post-hoc tests were performed with a Bonferroni correction

## Results

The mean RT for targets, non-targets, attended and unattended stimuli that were assessed before (pre-test) and after (post-test) the experimental protocols are shown separately for each PROTOCOL (EX and REST) in Supplementary Table 1

## *Targets/non-targets*

The analyses revealed a significant effect of TIME [F(1,23) = 27.29, p < .001, η2 = 0.54]. The average RT during the pre-test [M = 469 ms; SD = 119 ms] was significantly slower [p < .001] than for the post-test [M = 420; SD = 104 ms]. The TASK; PROTOCOL*TIME; TIME*TASK; PROTOCOL*TASK and PROTOCOL*TIME*TASK effects were non-significant.

## *Attended/unattended*

The analyses revealed a significant effect of TIME [F(1,23) = 35.68, p < .001, η2 = 0.61]. The average RT during the pre-test[M = 475 ms; SD = 119 ms] was significantly slower [p < .001] than for the post-test [M 422 ms=; SD = 103 ms]. The TASK; PROTOCOL*TIME; TIME*TASK; PROTOCOL*TASK and PROTOCOL*TIME*TASK effects were non-significant.

| **Supplementary Table 1.** Behavioral results – reaction times (ms) | | |
| --- | --- | --- |
|  | Pre-test  (M ± SD) | Post-test  (M ± SD) |
| EX protocol |  |  |
| TARGETS | 469 ± 121 | 423 ± 119 |
| NON-TARGETS | 483 ± 126 | 430 ± 117 |
| ATTENDED | 486 ± 130 | 435 ± 123 |
| UNATTENDED | 483 ± 130 | 428 ± 115 |
| REST protocol |  |  |
| TARGETS | 460 ± 127 | 412 ± 97 |
| NON-TARGETS | 463 ± 107 | 413 ± 84 |
| ATTENDED | 462 ± 112 | 415 ± 88 |
| UNATTENDED | 467 ± 104 | 410 ± 86 |
| *Note.* EX = vigorous-intensity exercise; REST = seated rest condition | | |

**SELECTIVE ATTENTION TASK**

**Task presentation parameters**

Please note that specific task parameters differed in comparison to Martinez et al. study^28^. Rather than presenting checkerboards for 100ms, we opted for a longer duration of 800ms. This modification allowed for analyses of brain responses in later time intervals and to avoid potential interactions with stimulus offset effects within the ERF time window of interest (0-600ms).

In Martinez's study, the low-frequency standards were set at 0.8 cpd (0.5 cpd for low-frequency targets), while the high-frequency standards were at 5 cpd (6 cpd for high-frequency targets). The difference between standards and targets was always kept constant. Instead, in our study, the target frequencies were adjusted on a trial-by-trial basis to maintain a consistent level of behavioral error, thereby aiming to keep attention at a constant level throughout the MEG session. Moreover, based on behavioral pilot studies (excluded from the final study), we determined that using standard/target stimuli of 0.8/0.5 cpd for low-frequency checkerboards and 1.5/2.8 cpd for high-frequency checkerboards allowed for a parallelized course of error rate adjustments across trials (i.e., parallel adjustments of difficulties for high and low frequencies). As such, low and high-frequency standard stimuli were more similar to each other than in the original study but still easily distinguishable.

It remains uncertain whether selecting the original parameters from Martinez's study would have altered any observed effects in our study.
